# Lipid metabolism abnormalities in pediatric abdominal solid malignant tumors: a comprehensive review

**DOI:** 10.3389/fped.2025.1727134

**Published:** 2025-12-17

**Authors:** Qiang Hao, Lu Han, Wenrui Mi, Tingting Hu, Aiqin Lin, Jie Liu

**Affiliations:** 1 Department of Pediatric Surgery, Yijishan Hospital of Wannan Medical College, Wannan Medical College, Wuhu, China; 2 Department of Medical Biology of Wannan Medical College, Wannan Medical College, Wuhu, China; 3 Department of Graduate School, Wannan Medical College, Wuhu, China; 4 Department of Science and Technology, Yijishan Hospital of Wannan Medical College, Wannan Medical College, Wuhu, China

**Keywords:** abdominal, lipid metabolism, pediatric, solid malignant tumor, update

## Abstract

Pediatric abdominal solid tumors mainly include neuroblastoma, nephroblastoma, hepatoblastoma, and rhabdomyosarcoma. Although significant progress has been made in the treatment of these tumors with the combination of surgery and chemotherapy, the treatment outcomes for some patients remain unsatisfactory, and the mortality rate for late-stage patients is still high. The diagnosis and treatment of abdominal solid tumors remain one of the key challenges in pediatric oncology. Numerous studies have found that lipid metabolism abnormalities in tumor tissues and cells lead to a significant increase in lipid levels, promoting cell proliferation, tumor progression, and chemoresistance. This review focuses on the recent advances in the study of lipid metabolism abnormalities in pediatric abdominal solid tumors, aiming to provide a theoretical basis for the treatment of these tumors.

## Introduction

Pediatric abdominal solid malignant tumors mainly include neuroblastoma (NB), nephroblastoma (WT), hepatoblastoma (HB), and rhabdomyosarcoma (RMS) (1–3). Despite the significant progress made in the treatment of these tumors with the combination of surgery and chemotherapy, the treatment outcomes for some patients remain unsatisfactory, and the mortality rate for late-stage patients is still high (4). Therefore, the diagnosis and treatment of abdominal solid tumors have become one of the key challenges in pediatric oncology and a focus of research for many scholars.

The regulation of lipid metabolism is crucial for maintaining cellular homeostasis (5). In normal cells, lipid metabolism-related genes are strictly regulated at the transcriptional level, keeping lipid synthesis at a low level (6). In recent years, metabolomics, an emerging discipline following genomics and proteomics, has gradually attracted the attention of scholars (7). Numerous studies have found that lipid metabolism abnormalities in tumor tissues and cells lead to a significant increase in lipid levels, promoting cell proliferation, tumor progression, and chemoresistance (8–10). With the deepening of research, some scholars have begun to focus on lipid metabolism abnormalities in pediatric tumors. This review focuses on the recent advances in the study of lipid metabolism abnormalities in pediatric abdominal solid tumors, aiming to provide a theoretical basis for the treatment of these tumors.

Although the four major pediatric abdominal solid malignant tumors described above each have distinct histological origins and clinical characteristics, accumulating evidence demonstrates significant commonalities in their lipid metabolism reprogramming. These tumors uniformly exhibit upregulated fatty acid synthase (FASN) expression, abnormal fatty acid oxidation, cholesterol metabolism dysregulation, and ferroptosis pathway imbalance. However, each tumor's metabolic vulnerability is uniquely shaped by its genetic background (e.g., MYCN amplification, Wnt/β-catenin pathway mutations) and microenvironmental differences. The following sections will elaborate on the specific metabolic abnormalities of each tumor, which will be further integrated in the Discussion to reveal both universal principles and individual variations in metabolic reprogramming of pediatric abdominal solid malignant tumors.

## Lipid metabolism abnormalities in NB

NB is a pediatric solid tumor. Studies have reported that approximately 150–200 children develop NB annually in Japan, with an incidence rate of about 7.7 per million in China, and its mortality rate accounts for 15% of the total mortality rate of pediatric tumors (11–13). Despite the use of various treatment methods, the 5-year survival rate for high-risk NB patients remains below 50%, indicating that the treatment of this disease still faces significant challenges (14). With the deepening of metabolomics research, scholars have recently conducted studies on lipid metabolism abnormalities in NB, providing important theoretical foundations for its treatment.

Tao et al. (13) found that MYCN can promote the accumulation of glycerolipids in NB. Researchers experimentally confirmed that MYCN can directly upregulate SLC27A2 to drive fatty acid uptake, which is essential for glycerolipid synthesis and MYCN-induced cell survival. NB driven by MYCN is believed to heavily rely on fatty acid uptake for survival, indicating that fatty acid uptake may be a promising therapeutic target for high-risk MYCN-amplified patients. Yu et al. (15) used bioinformatics methods to screen for genes related to lipid metabolism in NB and identified eight genes associated with lipid metabolism, namely ELOVL6, OSBPL9, RPL27A, HSD17B3, ACHE, AKR1C1, PIK3R1, and EPHX2. Further experiments confirmed that ELOVL6 promotes an immunosuppressive microenvironment and the malignant progression of NB. It is believed that these eight gene markers are significant in predicting the prognosis of NB, effectively classifying patients into high-risk and low-risk groups, and guiding innovative treatment strategies for these patients. Additionally, the signature gene ELOVL6 was found to significantly promote the immunosuppressive microenvironment and malignant progression of NB.

Wang et al. (14) developed an innovative sequential catalytic treatment system to intervene in the lipid metabolism and ferroptosis of NB, utilizing a non-crystalline zinc metal-organic framework (named LOx/HRP-aZIF) loaded with lactate oxidase (LOx) and horseradish peroxidase (HRP), in combination with the prodrug 3-indoleacetic acid (IAA). Based on the abnormal accumulation of lactate in the tumor microenvironment, the cascade reaction of LOx and HRP can consume endogenous glutathione and reduced nicotinamide adenine dinucleotide, achieving the first stage of killing cancer cells through antioxidant inactivation and disruption of the electron transport chain. Moreover, HRP and IAA can induce the production of reactive oxygen species through bioorthogonal catalysis, promoting ferritin degradation and lipid peroxidation, ultimately triggering self-enhancing ferroptosis and positive feedback by initiating the endogenous Fenton reaction. Freitas et al. (16) found that the accumulation of 7-dehydrocholesterol in tumor cells has a powerful pro-survival function. Since 7-dehydrocholesterol has excellent reactivity with peroxyl radicals, it can effectively protect (phospho)lipids from autoxidation and subsequent rupture. Researchers validated this in NB and Burkitt lymphoma xenografts, demonstrating that the accumulation of 7-dehydrocholesterol can induce these tumors to shift to an iron-resistant state, ultimately leading to a more aggressive phenotype. It is believed that the anti-ferroptotic activity of 7-dehydrocholesterol is an unrecognized intrinsic mechanism that cancer cells can exploit to evade ferroptosis and influence lipid metabolism disorders. Luo et al. (17) used bioinformatics to analyze the differences in lipid metabolism-related gene expression between high-risk and non-high-risk NB in the GSE49710 dataset, developing a prognostic model for NB based on lipid metabolism-related genes and demonstrating that GDPD5 can inhibit NB proliferation and migration and may be targeted and inhibited by hsa-miR-592 to inhibit fat synthesis in SH-SY5Y cells.

In summary, significant progress has been made in the study of lipid metabolism in NB. However, scholars also realize that the specific lipid metabolism disorders in NB may have very complex mechanisms of occurrence, and more in-depth studies with larger sample sizes may be needed in the future.

## Lipid metabolism abnormalities in nephroblastoma

Nephroblastoma is the most common primary malignant kidney tumor in children. As an embryonic tumor, it is also known as renal embryoma or Wilms tumor (WT) (18). Nephroblastoma accounts for 5% of all pediatric malignant tumors and 80% of all renal cancers diagnosed in children and adolescents (19). With the combination of surgery and adjuvant chemotherapy, the overall survival rate of this disease in children is about 90%. However, studies have reported that the recurrence rate of nephroblastoma remains as high as 15% (20). To solve this clinical dilemma, scholars have conducted various studies on the occurrence and development mechanisms of this disease. In recent years, the role of lipid metabolism in tumors has increasingly attracted attention, and important progress has also been made in the study of lipid metabolism in WT.

Wu et al. (21) used mass spectrometry to screen for differentially expressed proteins in nephroblastoma and adjacent normal tissues and validated the results through Western blot analysis. They found that hydroxyacyl-CoA dehydrogenase trifunctional multienzyme complex subunit alpha (a lipid metabolism enzyme, HADHA) is expressed at a lower level in nephroblastoma tissues. Additionally, the researchers found that the expression of HADHA is closely related to histopathological types, with high HADHA expression indicating poor prognosis. Wang et al. (22) conducted gene detection on three cases of nephroblastoma tissues and adjacent normal tissues to screen differentially expressed miRNAs (DEMs). They identified three miRNAs related to lipid metabolism targeting the FASN gene, namely miR-107, miR-27a-3p, and miR-335-5p. Further experimental validation showed that miR-27a-3p is significantly associated with nephroblastoma. It is believed that the imbalanced expression of miRNAs may participate in the occurrence and development of nephroblastoma through lipid metabolism. The expression of miR-27a-3p is related to the malignancy of nephroblastoma and may become a target for the diagnosis, prognosis, and treatment of nephroblastoma in the future. Tasic et al. (23) collected 14 nephroblastoma samples and seven normal kidney tissue samples and conducted metabolomics studies on nephroblastoma using high-resolution magic-angle spinning nuclear magnetic resonance spectroscopy. The results showed that the concentrations of glutamine/glutamate, lipids, and lactate metabolites were significantly increased in nephroblastoma, while the concentration of branched-chain amino acid metabolites was decreased. It is believed that this finding opens up prospects for further research and clinical validation of nephroblastoma. Wang et al. (24) conducted proteomics analysis to detect lipid metabolism enzymes in nine tissue samples from nephroblastoma and adjacent tissues. Proteomics revealed 19 differentially expressed lipid metabolism enzymes. Protein-protein interaction analysis was used to identify interacting proteins. Immunohistochemistry, immunofluorescence, and Western blot were used to further confirm whether the expression of fatty acid synthase (FASN) was significantly increased in tumor tissues. The results showed that high expression of FASN in nephroblastoma is associated with poor prognosis, and it is believed that FASN can serve as a prognostic biomarker for nephroblastoma patients.

The above studies all suggest that lipid metabolism plays an important role in the occurrence and development of nephroblastoma, and some studies have even identified biomarkers closely related to the prognosis of nephroblastoma. However, most of the current research is still in the basic research stage, and there are few clinical research experiments on drugs that adjust lipid metabolism for the treatment of nephroblastoma. More efforts from subsequent scholars are needed.

## Lipid metabolism abnormalities in HB

HB is the most common malignant liver tumor in children, predominantly occurring in children under the age of 3, with an incidence rate of approximately 1 per million (25). The pathogenesis of this disease remains unclear. With the combined application of neoadjuvant chemotherapy and surgery, the survival rate of HB has increased from 40% to over 70%. However, the treatment outcomes for late-stage HB patients are still poor, and recurrence remains a significant clinical challenge (26, 27). Additionally, targeted therapy for this disease has not made significant progress, which continues to perplex scholars. Li et al. (28) used a gene knockout animal model to discover that SETD2 deficiency downregulates the expression of H3K36me3-enriched and cholesterol efflux genes, leading to lipid accumulation. A high-fat diet increased lipid accumulation and promoted liver cancer development in Setd2-deficient mice. Hu et al. (29) found that the mutual inhibition of FoxO1 and SREBP-1c regulates the progression of HB by modulating fatty acid metabolism. Alannan et al. (30) discovered that PCSK9 deficiency strongly inhibits cell proliferation in all HB cell lines. At the lipid metabolism level, the inhibitory effect of PCSK9 is achieved through an increase in intracellular neutral lipids, phospholipids, and polyunsaturated fatty acids, as well as the accumulation of lipid peroxides. Liu et al. (31) discovered that SLC7A11, a critical lipid metabolism-related transporter, is highly upregulated in hepatoblastoma (HB) and promotes tumorigenesis by enhancing ferroptosis resistance. They found that the m6A modification mediated by METTL3 and recognized by IGF2BP1 stabilizes SLC7A11 mRNA through inhibiting deadenylation, thereby increasing its expression. These findings suggest that targeting the m6A-SLC7A11 axis may represent a potential therapeutic strategy for HB. Huang et al. (32) found that silencing the FASN gene can lead to lipid metabolism abnormalities in HB cells, thereby inhibiting the proliferation and migration of HB cells and inducing HB cell apoptosis. In summary, an increasing number of studies have shown that lipid metabolism disorders exist in the occurrence and development of HB. Focusing on targeting the abnormal lipid metabolism of HB tumors has become a new anticancer research strategy and a hotspot for scholars in recent years (33).

## Lipid metabolism abnormalities in RMS

RMS is the most common malignant tumor of pediatric soft tissues, originating from myogenic mesenchymal progenitor cells and occurring in various organs and tissues such as the bladder, gonads, nasopharynx, paranasal sinuses, meninges, and orbit (34, 35). Although the combination of various treatment methods has improved the prognosis for some RMS patients, treatment-related toxicity leading to organ damage has also become a serious clinical issue (36, 37). In recent years, some scholars have also conducted research on the lipid metabolism and related pathogenesis of RMS. Zhang et al. (38) discovered that PLIN2 is widely expressed in RMS, with significant differences compared to other non-lipomatous sarcomas. This suggests that PLIN2 may play a crucial role in lipid metabolism regulation within RMS, potentially influencing tumor differentiation and progression. The high expression of PLIN2 could be associated with the abnormal lipid accumulation observed in these tumors, contributing to their unique metabolic profile. Further investigation into the specific mechanisms by which PLIN2 affects lipid metabolism may provide insights into targeted therapeutic strategies for RMS. Milosevic et al. (39) investigated the antitumor activity of the natural pigment violacein in osteosarcoma (OS) and RMS cell lines. They found that violacein exhibited selective toxicity towards these cancer cells, with IC50 values ranging from 0.35 to 0.88 µM. The compound induced apoptosis and inhibited cell migration in both OS and RMS cells. Mechanistically, violacein acted independently of oxidative signaling, as it did not increase intracellular ROS generation or lipid peroxidation. The study demonstrated that violacein has potential as an anticancer agent for OS and RMS, warranting further investigation into its therapeutic applications. Miyagaki et al. (40) inhibited lipid metabolism in RMS cells *in vitro* by administering a propionyl-CoA carboxylase inhibitor, showing that the inhibition of lipid metabolism disrupted the cancer-specific metabolic balance in RMS, thereby producing a tumor growth inhibitory effect. Dächert et al. (41) found that RMS is susceptible to Erastin-induced cell death. Erastin, as an inhibitor of the glutamate/cystine antiporter xc-, can increase reactive oxygen species (ROS) production through glutathione (GSH) depletion. Before cell death, Erastin can cause GSH exhaustion, ROS production, and lipid peroxidation. This study provides new insights into the molecular mechanisms of ferroptosis and lipid peroxidation in RMS, which is conducive to the development of new redox-based therapeutic strategies.

## Conclusions and future perspectives

**Figure 1:**
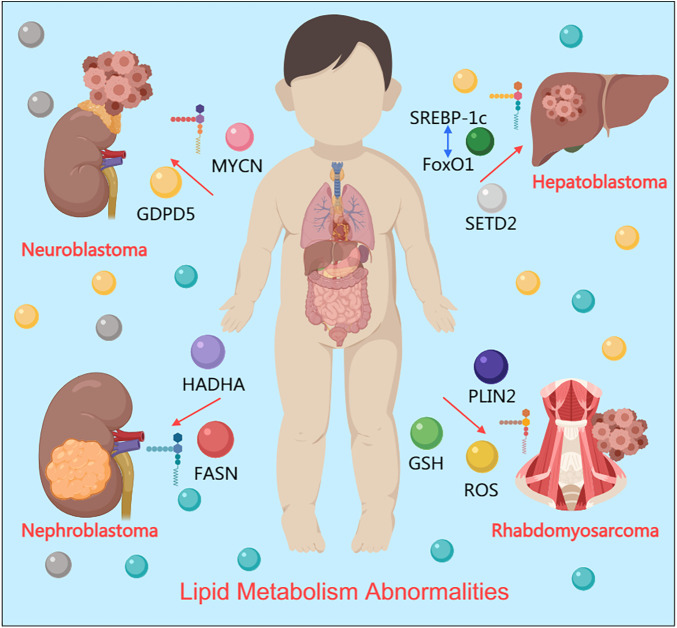
Lipid metabolism abnormalities in pediatric abdominal solid malignant tumors.

In recent years, domestic and international scholars have conducted a large number of studies on lipid metabolism abnormalities in pediatric abdominal solid malignant tumors. The results show that lipid metabolism disorders in pediatric abdominal tumors are closely related to tumor occurrence, development, invasion, and metastasis (Figure 1). Lipid metabolism pathways and related enzymes in tumors may be potential targets for anticancer drug therapy. These studies not only provide important supplements to the mechanisms of occurrence and development of pediatric abdominal solid malignant tumors but also provide important theoretical basis for the nutritional support treatment of pediatric abdominal tumors.

The lipid metabolism abnormalities in different tumors are closely associated with their specific pathological features. In neuroblastoma, MYCN-driven enhancement of fatty acid uptake not only satisfies the energy demands of rapid proliferation but also promotes chemoresistance through the generation of specific membrane phospholipids, a mechanism particularly pronounced in high-risk patients. In Wilms tumor, high FASN expression is associated with increased tumor recurrence risk, possibly by maintaining stem cell-like properties, which provides a metabolic explanation for its high recurrence rate of up to 15%. In hepatoblastoma, cholesterol metabolism dysregulation synergizes with Wnt/β-catenin pathway activation to promote tumor initiation, as validated in SETD2-deficient models. In rhabdomyosarcoma, PLIN2 overexpression maintains intracellular redox homeostasis through lipid droplet formation, supporting invasive growth that aligns with its frequent occurrence in anatomically complex regions such as the head and neck. Although these associations are partially based on mechanistic speculation, they provide important clues for understanding the causal role of metabolic reprogramming in tumor progression and lay a theoretical foundation for developing individualized treatment strategies.

However, we also need to recognize that the occurrence mechanisms of tumors are very complex, and more scholars are needed to conduct related cell, animal, and clinical drug studies in the future, which may provide more precise treatment methods for pediatric abdominal solid malignant tumors.
